# Post-discharge kidney function is associated with subsequent ten-year renal progression risk among survivors of acute kidney injury

**DOI:** 10.1016/j.kint.2017.02.019

**Published:** 2017-08

**Authors:** Simon Sawhney, Angharad Marks, Nick Fluck, Adeera Levin, David McLernon, Gordon Prescott, Corri Black

**Affiliations:** 1 University of Aberdeen, Aberdeen, UK; 2 NHS Grampian, Aberdeen, UK; 3 University of British Columbia, British Columbia, Canada

**Keywords:** acute kidney injury, chronic kidney disease, epidemiology, mortality, progression, prognosis

## Abstract

The extent to which renal progression after acute kidney injury (AKI) arises from an initial step drop in kidney function (incomplete recovery), or from a long-term trajectory of subsequent decline, is unclear. This makes it challenging to plan or time post-discharge follow-up. This study of 14651 hospital survivors in 2003 (1966 with AKI, 12685 no AKI) separates incomplete recovery from subsequent renal decline by using the post-discharge estimated glomerular filtration rate (eGFR) rather than the pre-admission as a new reference point for determining subsequent renal outcomes. Outcomes were sustained 30% renal decline and de novo CKD stage 4, followed from 2003-2013. Death was a competing risk. Overall, death was more common than subsequent renal decline (37.5% vs 11.3%) and CKD stage 4 (4.5%). Overall, 25.7% of AKI patients had non-recovery. Subsequent renal decline was greater after AKI (vs no AKI) (14.8% vs 10.8%). Renal decline after AKI (vs no AKI) was greatest among those with higher post-discharge eGFRs with multivariable hazard ratios of 2.29 (1.88-2.78); 1.50 (1.13-2.00); 0.94 (0.68-1.32) and 0.95 (0.64-1.41) at eGFRs of 60 or more; 45-59; 30-44 and under 30, respectively. The excess risk after AKI persisted over ten years of study, irrespective of AKI severity, or post-episode proteinuria. Thus, even if post-discharge kidney function returns to normal, hospital admission with AKI is associated with increased renal progression that persists for up to ten years. Follow-up plans should avoid false reassurance when eGFR after AKI returns to normal.

Acute kidney injury (AKI) is common and associated with poor renal outcomes,^1^ but the clinical course is not well understood.^2^, ^3^, ^4^ One reason for the increase in advanced chronic kidney disease (CKD) after AKI (vs. no AKI) is “nonrecovery,” that is, the occurrence of a step drop in estimated glomerular filtration rate (eGFR) during the AKI episode, which does not return to baseline once the episode has ended (Figure 1, pink dashed line). However, another path to advanced CKD after AKI may be a trajectory of *subsequent* renal decline after the episode has ended (Figure 1, red solid line). This distinction between subsequent progression and nonrecovery is crucial in clinical practice. At the time of a post-discharge clinical review, future subsequent renal decline is uncertain, whereas the extent of nonrecovery can already be observed. Moreover, because the trajectory of renal decline can vary from a gradual to a catastrophic loss of function,^5^ both hard outcomes (e.g., *de novo* long-term renal replacement therapy [RRT] or CKD stage 4) and intermediate outcomes (e.g., a 30% drop in kidney function)^6^ are important for clinicians and their patients to understand when planning care.

**Figure 1:**
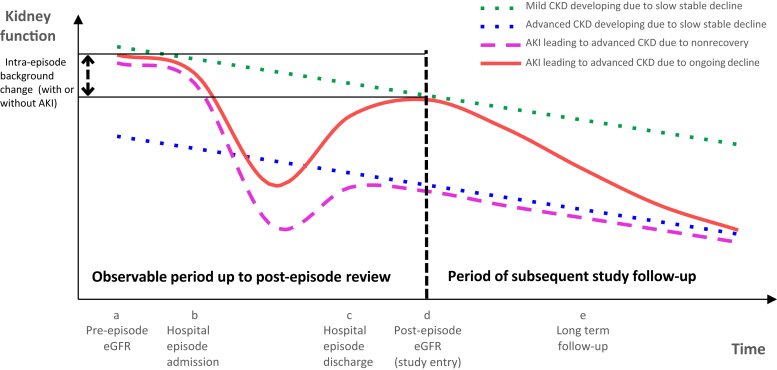
**Renal progression after acute kidney injury (AKI) caused by renal decline (red solid line) or nonrecovery (pink dashed line).** A patient with AKI who has incomplete post-episode recovery has a high risk of developing advanced chronic kidney disease (CKD) even if *subsequent* renal decline is slow (pink dashed line). However, the risk of advanced CKD in a patient with AKI who has near-complete recovery depends on whether he or she experiences *subsequent* decline at a rapid trajectory (red solid line). In both cases at a post-AKI reassessment review (time d), renal recovery and post-episode kidney function are already observable, but the risk of subsequent decline is uncertain. The vertical black dashed line at time d represents the start of follow-up in this study. eGFR, estimated glomerular filtration rate.

The Kidney Disease: Improving Global Outcomes AKI guidelines provide advice for post-AKI management based on expert opinion but without graded evidence.^7^ They state that people with AKI should be re-evaluated for resolution of kidney function and receive care based on CKD guidelines if they have developed CKD. However, this guidance does not apply to those who have had an episode of AKI and recovered to normal levels of kidney function after the episode. The relevance of post-episode recovery to baseline as a stratifying risk factor for AKI outcomes has been previously recognized in some studies but not in others.^8^ This is because previous studies have dichotomized recovery as being present or absent, with each using different cutoff values, but in reality, a spectrum of renal recovery exists.^9^, ^10^ Moreover, even if patients could be adequately grouped by recovery status, the use of a pre-episode baseline for determining outcomes would not separate the initial progression caused by incomplete recovery (however slight) from the subsequent progression caused by ongoing decline. The solution in clinical practice is that a clinician will wait to see where a post-episode eGFR finally settles (which becomes the “new baseline”) before evaluating risk and planning care from that point on. Therefore this is the approach we adopted in our analysis.

In this large population study, we evaluated whether a completed AKI episode was still associated with subsequent renal decline, after allowing for a variable extent of initial renal recovery to baseline once the episode has ended. We isolated subsequent renal decline by using post-episode eGFR as the reference for subsequent renal outcomes. We hypothesized that more patients with AKI (vs. no AKI) would experience ongoing renal decline (a 30% eGFR drop), resulting in more patients with AKI having CKD stage 4.

## Results

### Population

**Figure 2:**
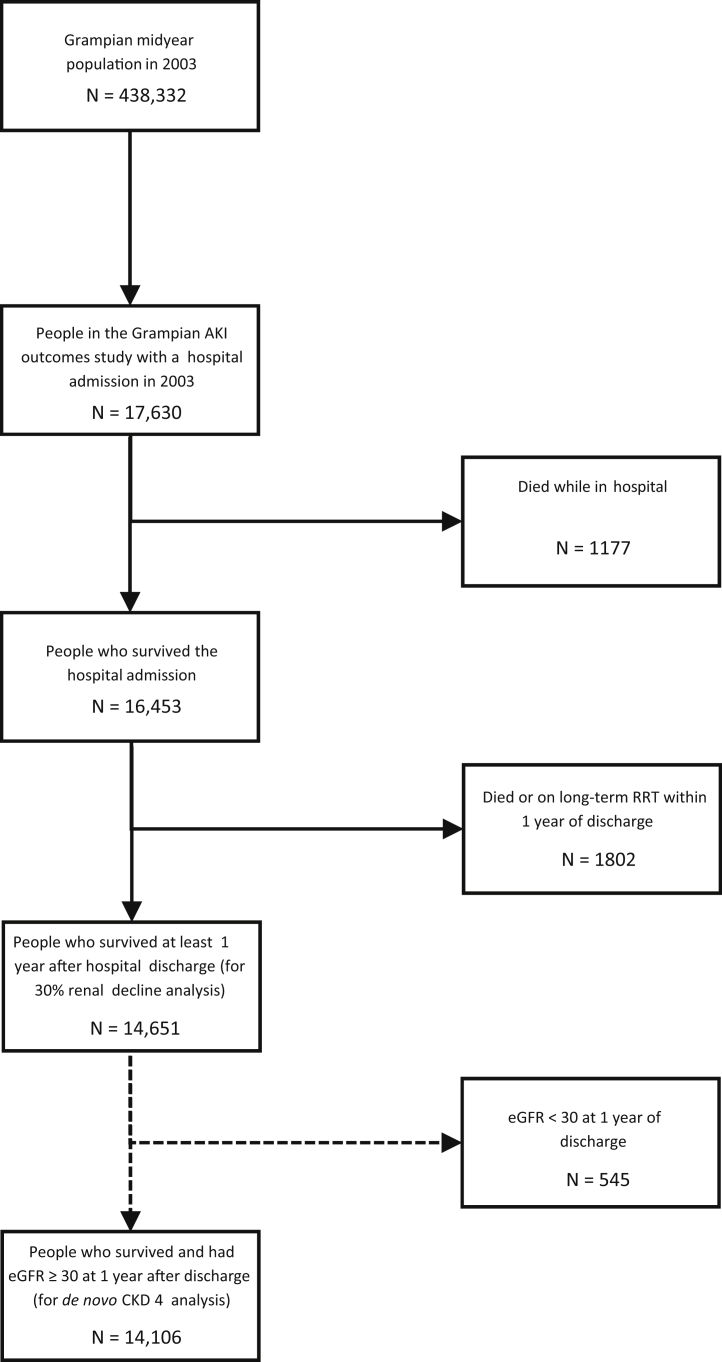
**Flow diagram showing derivation of the cohort from the Grampian population.** AKI, acute kidney injury; RRT, renal replacement therapy.

Of 17,630 patients with an index hospital admission in 2003, 14,651 patients were alive and not receiving long-term RRT 1 year after index hospital admission (Figure 2). This included 1966 with AKI and 12,685 without AKI. For the study of *de novo* CKD stage 4, an additional 545 patients who already had eGFR < 30 ml/min per 1.73 m^2^ at study entry were excluded. Thus 14,651 patients were available for the study of renal decline and 14,106 for the study of *de novo* CKD stage 4. The follow-up period of the study extended up to 10 years after the hospital admission, including 93,419 patient-years and a median of 8.6 years of follow-up.

### Key findings

From a reference point of the eGFR 1 year after a hospital admission episode, sustained subsequent 30% eGFR decline developed in 1660 of 14,651 patients (11.3%; 14.8% for AKI, 10.8% for no AKI), and sustained new CKD stage 4 occurred in 632 of 14,106 patients (4.5%; 7.1% for AKI, 4.1% for no AKI). Overall, patients were more likely to die than experience subsequent renal progression, whether defined as 30% renal decline (37.5% vs. 11.3%) or as new CKD stage 4 (38.8% vs. 4.5%).

### Characteristics of patients with and without AKI

Table 1 describes the characteristics of patients with and without AKI during the index hospital admission. Those with AKI (vs. no AKI) were older and were more frequently admitted on an emergency basis or to a critical care setting. They had more comorbidities. Although pre-hospital episode baseline eGFR was higher among those with AKI, post-discharge eGFR was lower, and a greater proportion of patients had a 30% decline in eGFR from the pre-hospital episode to the post-hospital episode (25.7% AKI, 2.3% no AKI) (i.e., nonrecovery). Nonrecovery was especially common among patients with AKI and post-episode eGFR < 60 ml/min per 1.73 m^2^ (42.3%). The proportion of patients with post-episode proteinuria was also 3-fold higher among those with AKI.

**Table 1:** Baseline characteristics for patients with and without acute kidney injury

Characteristic	Overall		AKI		No AKI	
	N	%	N	%	N	%
N	14,651		1966	(13.4% of cohort)	12,685	(86.6% of cohort)
Age in years (median & IQR)	69	(54–78)	73	(63–81)	68	(53–78)
Female	8317	(56.8)	1011	(51.4)	7306	(57.6)
Residential care	433	(3.0)	111	(5.6)	322	(2.5)
Deprived home location^a^	1215	(8.3)	169	(8.6)	1046	(8.2)
Rural home location	4014	(27.4)	551	(28.0)	3463	(27.3)
Emergency hospital admission	8689	(59.3)	1580	(80.4)	7109	(56.0)
Medical specialty admission	7203	(49.2)	1336	(68.0)	5867	(46.3)
Critical care admission	1288	(8.8)	529	(26.9)	759	(6.0)
Intensive care admission	428	(2.9)	276	(14.0)	152	(1.2)
Length of hospital stay in days (median & IQR)	3	(1–9)	14	(7–31)	2	(1–7)
Cancer	1011	(6.9)	201	(10.2)	810	(6.4)
Cardiac failure	668	(4.6)	181	(9.2)	487	(3.8)
Cerebrovascular disease	613	(4.2)	124	(6.3)	489	(3.9)
Dementia	150	(1.0)	30	(1.5)	120	(0.9)
Diabetes	917	(6.3)	255	(13.0)	662	(5.2)
Liver disease	189	(1.3)	49	(2.5)	140	(1.1)
Myocardial infarction	735	(5.0)	182	(9.3)	553	(4.4)
Neurologic disease	76	(0.5)	20	(1.0)	56	(0.4)
Peptic disease	304	(2.1)	66	(3.4)	238	(1.9)
Peripheral vascular disease	487	(3.3)	140	(7.1)	347	(2.7)
Pulmonary disease	836	(5.7)	199	(10.1)	637	(5.0)
Rheumatic disease	312	(2.1)	68	(3.5)	244	(1.9)
Baseline (pre-episode) eGFR (median & IQR)	66.8	(53.0–88.2)	75.3	(53.9–91.8)	65.8	(52.9–87.3)
Post-episode eGFR^b^						
≥60	9004	(61.5)	955	(48.6)	8049	(63.5)
45–59	3369	(23.0)	444	(22.6)	2925	(23.1)
30–44	1733	(11.8)	374	(19.0)	1359	(10.7)
<30	545	(3.7)	193	(9.8)	352	(2.8)
Intra-episode background change in eGFR^c^						
>30% rise	1135	(7.7)	67	(3.4)	1068	(8.4)
10%–30% rise	2069	(14.1)	120	(6.1)	1949	(15.4)
No change	7654	(52.2)	517	(26.3)	7137	(56.3)
10%–30% fall	2990	(20.4)	757	(38.5)	2233	(17.6)
>30% fall	803	(5.5)	505	(25.7)	298	(2.3)
Post-episode proteinuria^d^						
Untested	13069	(89.2)	1550	(78.8)	11519	(90.8)
Normal	753	(5.1)	136	(6.9)	617	(4.9)
Abnormal	829	(5.7)	280	(14.2)	549	(4.3)
AKI stage						
0	12685	(86.6)	n/a	–	12685	(100.0)
1	1355	(9.2)	1355	(68.9)	n/a	–
2	410	(2.8)	410	(20.9)	n/a	–
3	201	(1.4)	201	(10.2)	n/a	–
Prior AKI episodes	1358	(9.3)	356	(18.1)	1002	(7.9)

AKI, acute kidney injury; eGFR, estimated glomerular filtration rate; IQR, interquartile range; n/a, not applicable.

a Most deprived quintile of the Scottish Index of Multiple Deprivation.

b Post-episode eGFR was the most recent available eGFR at a time point 1 year after discharge from the index hospital admission. This was used as the reference for determining subsequent renal outcomes.

c Intra-episode background change in eGFR was the change between pre-episode baseline and post-episode eGFR (that can occur irrespective of the presence of AKI).

d Post-episode proteinuria was based on proteinuria measurements taken during or within 1 year of the index hospital admission.

### Crude proportions and cumulative rates of subsequent renal progression

**Figure 3:**
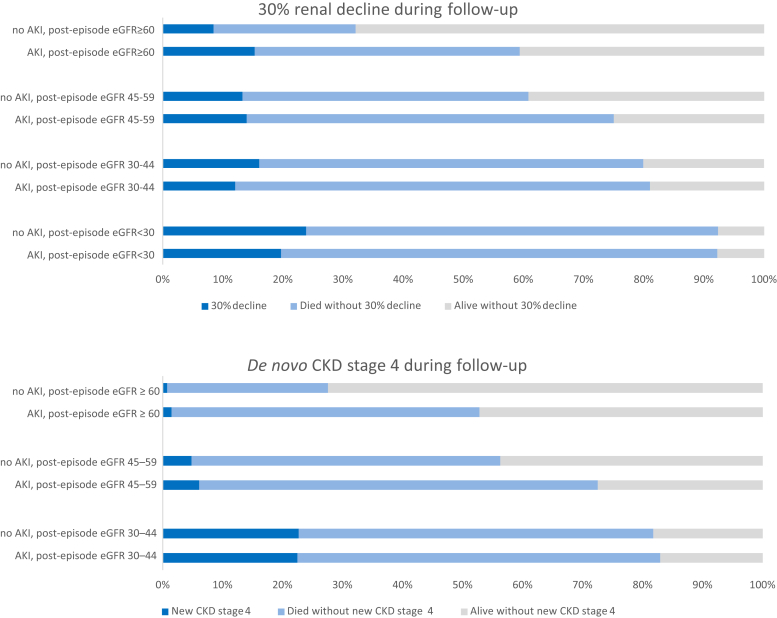
**Crude long-term renal outcomes after a hospital admission episode with or without acute kidney injury (AKI).** CKD, chronic kidney disease; eGFR, estimated glomerular filtration rate.

Figure 3 shows the crude proportions of people alive 1 year after hospital discharge who subsequently experienced renal decline (top plot), *de novo* CKD stage 4 (bottom plot), and death before progression during the study follow-up period until 10 years after discharge. After AKI (vs. no AKI), there was an excess of renal decline, or decline and death outcomes combined, among those with post-episode eGFR ≥ 60 ml/min per 1.73 m^2^. This excess was not present among those with a lower post-episode eGFR. The outcome of *de novo* CKD stage 4 was also more common among those with AKI, but uncommon among those with post-episode eGFR ≥ 60 ml/min per 1.73 m^2^ (1.5% AKI, 0.7% no AKI) and eGFR 45–59 ml/min per 1.73 m^2^ (6.1% AKI, 4.8% no AKI), compared with eGFR 30–44 ml/min per 1.73 m^2^ (22.5% AKI, 22.7% no AKI). Supplementary Figure S1 shows crude outcomes at 5 years after hospital discharge, with similar relationships, but with fewer deaths.

**Figure 4:**
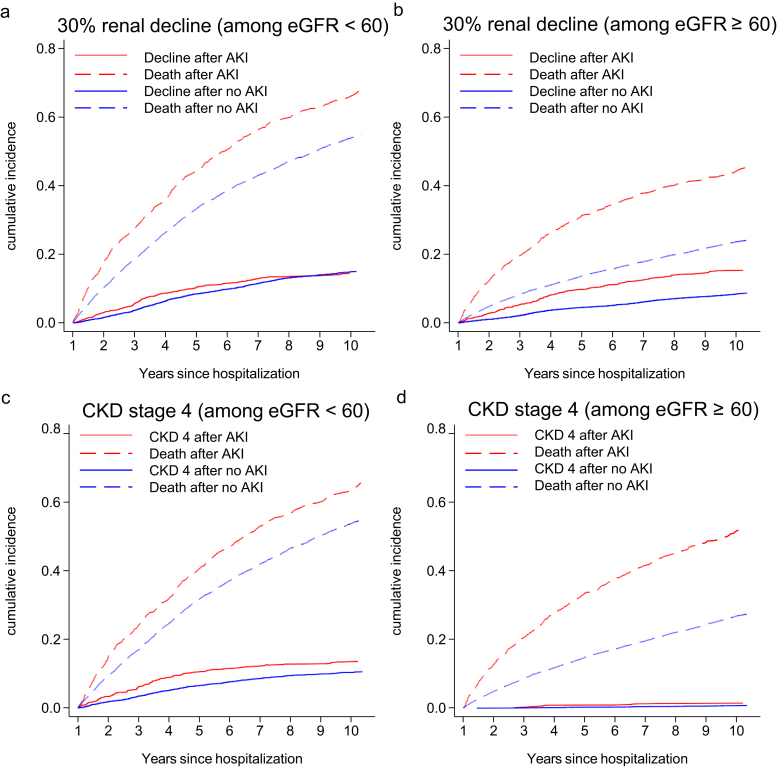
**Cumulative incidences of subsequent renal progression (solid line) for those with (red) and without (blue) an acute kidney injury (AKI) admission in 2003, grouped by postdischarge estimated glomerular rate (eGFR) and accounting for the competing risk of death (dashed line).** (**a**,**b**) Subsequent sustained 30% renal decline; (**c**,**d**) new chronic kidney disease (CKD) stage 4.

Figure 4 shows the cumulative incidences of subsequent renal decline (a and b) and *de novo* CKD stage 4 (c and d) stratified by AKI and post-episode eGFR, accounting for the competing risk of death. Follow-up in all plots starts 1 year after discharge (i.e., study entry). Death without progression was more common than either progression outcome, and in the absence of a post-episode eGFR < 60 ml/min per 1.73 m^2^, *de novo* CKD stage 4 was rare.

### Characteristics of patients with and without subsequent renal progression

Table 2 describes the baseline characteristics of patients who progressed, died, or were alive without subsequent progression at the end of the study. Those with subsequent progression by either definition were older and had more comorbidities, including baseline renal impairment, as were those who died. A greater proportion of patients who experienced progression had diabetes as compared with those who died without progression, whereas the reverse was true for the other comorbidities.

**Table 2:** Baseline characteristics for each progression group

Characteristic	Overall		No renal decline or death		New sustained 30% renal decline		Dead before 30% renal decline		No CKD stage 4 or death		*De novo* CKD stage 4		Dead before *de novo* CKD stage 4	
	N	%	N	%	N	%	N	%	N	%	N	%	N	%
N	14,651		7497	(51.2% of cohort)	1660	(11.3% of cohort)	5494	(37.5% of cohort)	7996	(56.7% of cohort)	632	(4.5% of cohort)	5478	(38.8% of cohort)
Age in years (median & IQR)	69	(54–78)	57	(42–69)	73	(65–79)	78	(71–84)	58	(43–69)	75	(69–82)	78	(70–84)
Female	8317	(56.8)	4168	(55.6)	959	(57.8)	3190	(58.1)	4485	(56.1)	353	(55.9)	3150	(57.5)
Residential care	433	(3.0)	36	(0.5)	25	(1.5)	372	(6.8)	36	(0.5)	11	(1.7)	351	(6.4)
Deprived home location^a^	1215	(8.3)	624	(8.3)	126	(7.6)	465	(8.5)	646	(8.1)	50	(7.9)	476	(8.7)
Rural home location	4014	(27.4)	2137	(28.5)	447	(26.9)	1430	(26.0)	2274	(28.4)	158	(25.0)	1432	(26.1)
Emergency hospital admission	8689	(59.3)	4027	(53.7)	939	(56.6)	3723	(67.8)	4281	(53.5)	371	(58.7)	3653	(66.7)
Medical specialty admission	7203	(49.2)	3158	(42.1)	880	(53.0)	3165	(57.6)	3399	(42.5)	346	(54.7)	3139	(57.3)
Critical care admission	1288	(8.8)	634	(8.5)	166	(10.0)	488	(8.9)	692	(8.7)	63	(10.0)	482	(8.8)
Intensive care admission	428	(2.9)	217	(2.9)	60	(3.6)	151	(2.7)	236	(3.0)	20	(3.2)	160	(2.9)
Cancer	1011	(6.9)	302	(4.0)	119	(7.2)	590	(10.7)	330	(4.1)	52	(8.2)	579	(10.6)
Cardiac failure	668	(4.6)	113	(1.5)	100	(6.0)	455	(8.3)	120	(1.5)	53	(8.4)	408	(7.4)
Cerebrovascular disease	613	(4.2)	113	(1.5)	85	(5.1)	415	(7.6)	126	(1.6)	36	(5.7)	402	(7.3)
Dementia	150	(1.0)	6	(0.1)	8	(0.5)	136	(2.5)	6	(0.1)	5	(0.8)	128	(2.3)
Diabetes	917	(6.3)	242	(3.2)	183	(11.0)	492	(9.0)	271	(3.4)	92	(14.6)	453	(8.3)
Liver disease	189	(1.3)	72	(1.0)	25	(1.5)	92	(1.7)	75	(0.9)	9	(1.4)	97	(1.8)
Myocardial infarction	735	(5.0)	226	(3.0)	96	(5.8)	413	(7.5)	241	(3.0)	60	(9.5)	379	(6.9)
Neurologic disease	76	(0.5)	21	(0.3)	9	(0.5)	46	(0.8)	21	(0.3)	5	(0.8)	47	(0.9)
Peptic disease	304	(2.1)	103	(1.4)	46	(2.8)	155	(2.8)	111	(1.4)	17	(2.7)	157	(2.9)
Peripheral vascular disease	487	(3.3)	113	(1.5)	80	(4.8)	294	(5.4)	118	(1.5)	40	(6.3)	273	(5.0)
Pulmonary disease	836	(5.7)	229	(3.1)	108	(6.5)	499	(9.1)	245	(3.1)	46	(7.3)	512	(9.3)
Rheumatic disease	312	(2.1)	91	(1.2)	37	(2.2)	184	(3.3)	106	(1.3)	14	(2.2)	177	(3.2)
Baseline (pre-episode) eGFR (median & IQR)	66.8	(53.0–88.2)	79.6	(61.8–98.7)	57.7	(46.2–70.5)	57.3	(45.8–73.7)	78.8	(61.6–97.9)	45.8	(37.4–55.4)	59.5	(49.0–75.4)

There are 14,106 patients in *de novo* CKD stage 4 analysis because 545 patients had eGFR<30 at study entry.

AKI, acute kidney injury; eGFR, estimated glomerular filtration rate; IQR, interquartile range.

a Most deprived quintile of the Scottish Index of Multiple Deprivation.

Table 3 describes renal measurements within each progression group. Those with subsequent progression by either definition had a greater proportion with a low post-episode eGFR, proteinuria, AKI, prior AKI, and *any* change in eGFR (whether a rise or fall) during the admission episode. The proportion with post-episode proteinuria was 2-fold higher in those with progression than in those who died without progression. The increased progression among those with AKI did not vary by AKI stage.

**Table 3:** Renal measurements for each progression group

Renal measurement	Overall		No renal decline or death		New sustained 30% renal decline		Dead before 30% renal decline		No CKD stage 4 or death		*De novo* CKD stage 4		Dead before *de novo* CKD stage 4	
	N	%	N	%	N	%	N	%	N	%	N	%	N	%
n	14,651		7497	(51.2% of cohort)	1660	(11.3% of cohort)	5494	(37.5% of cohort)	7996	(56.7% of cohort)	632	(4.5% of cohort)	5478	(38.8% of cohort)

**Post-episode eGFR**^a^														
≥60	9004	(61.5)	5854	(78.1)	825	(49.7)	2325	(42.3)	6282	(78.6)	73	(11.6)	2649	(48.4)
45–59	3369	(23.0)	1257	(16.8)	450	(27.1)	1662	(30.3)	1402	(17.5)	167	(26.4)	1800	(32.9)
30–44	1733	(11.8)	344	(4.6)	263	(15.8)	1126	(20.5)	312	(3.9)	392	(62.0)	1029	(18.8)
<30	545	(3.7)	42	(0.6)	122	(7.3)	381	(6.9)						

**Intra-episode background change in eGFR**^b^														
>30% rise	1135	(7.7)	420	(5.6)	226	(13.6)	489	(8.9)	498	(6.2)	45	(7.1)	572	(10.4)
10%–30% rise%	2069	(14.1)	923	(12.3)	277	(16.7)	869	(15.8)	1014	(12.7)	102	(16.1)	909	(16.6)
No change	7654	(52.2)	4659	(62.1)	703	(42.3)	2292	(41.7)	4933	(61.7)	243	(38.4)	2344	(42.8)
10%–30% fall	2990	(20.4)	1282	(17.1)	362	(21.8)	1346	(24.5)	1346	(16.8)	179	(28.3)	1302	(23.8)
>30% fall	803	(5.5)	213	(2.8)	92	(5.5)	498	(9.1)	205	(2.6)	63	(10.0)	351	(6.4)

**Post-episode proteinuria**^c^														
Untested	13069	(89.2)	6985	(93.2)	1302	(78.4)	4782	(87.0)	7416	(92.7)	469	(74.2)	4809	(87.8)
Normal	753	(5.1)	340	(4.5)	122	(7.3)	291	(5.3)	386	(4.8)	51	(8.1)	291	(5.3)
Abnormal	829	(5.7)	172	(2.3)	236	(14.2)	421	(7.7)	194	(2.4)	112	(17.7)	378	(6.9)

**AKI stage**														
0	12685	(86.6)	6912	(92.2)	1369	(82.5)	4404	(80.2)	7359	(92.0)	507	(80.2)	4467	(81.5)
1	1355	(9.2)	387	(5.2)	201	(12.1)	767	(14.0)	432	(5.4)	86	(13.6)	711	(13.0)
2	410	(2.8)	124	(1.7)	57	(3.4)	229	(4.2)	128	(1.6)	26	(4.1)	219	(4.0)
3	201	(1.4)	74	(1.0)	33	(2.0)	94	(1.7)	77	(1.0)	13	(2.1)	81	(1.5)
Prior AKI episodes	1358	(9.3)	355	(4.7)	230	(13.9)	773	(14.1)	379	(4.7)	110	(17.4)	705	(12.9)

There are 14,106 patients in *de novo* CKD stage 4 analysis because 545 patients had eGFR<30 at study entry.

AKI, acute kidney injury; CI, confidence interval; eGFR, estimated glomerular filtration rate.

a Post-episode eGFR was the most recent available eGFR at a time point 1 year after discharge from the index hospital admission. This was used as the reference for determining subsequent renal outcomes.

b Intra-episode background change in eGFR was the change between pre-episode baseline and post-episode eGFR (that can occur irrespective of the presence of AKI).

c Post-episode proteinuria was based on proteinuria measurements taken during or within 1 year of the index hospital admission.

### Independent association between AKI and subsequent renal progression

Table 4 describes the multivariable-adjusted relationship between AKI and renal decline stratified by post-episode eGFR (interaction *P* < 0.001). The plain text indicates relative risks compared with a reference group with no AKI and eGFR ≥ 60 ml/min per 1.73 m^2^. Bold text indicates AKI versus no AKI at each level of post-episode eGFR. The relative risk of renal decline for AKI (vs. no AKI) was greater in those with otherwise normal function than in those with lower post-episode eGFR: hazard ratio (HR) (for AKI vs. no AKI) 2.29 (1.88–2.41), 1.50 (1.13–2.00), 0.84 (0.68–1.32) and 0.95 (0.64–1.41) for post-episode eGFR ≥ 60, 45–59, 30–45, and <30 ml/min per 1.73 m^2^, respectively. Table 5 shows a similar relationship between AKI and *de novo* CKD stage 4, but the magnitude of the variation with post-episode eGFR was greater.

**Table 4:** Relative risk of subsequent sustained 30% renal decline after acute kidney injury

Post-hospital episode eGFR	AKI or no AKI	N	Cause-specific renal decline; age-sex adjusted (HR, 95% CI)	Cause-specific renal decline; fully adjusted (HR, 95% CI)	Competing event of death without renal decline; age-sex adjusted (HR, 95% CI)	Competing event of death without renal decline; fully adjusted (HR, 95% CI)
eGFR ≥ 60	No AKI (reference)	8049	1.00 (reference)	1.00 (reference)	1.00 (reference)	1.00 (reference)
	AKI	955	2.01 (1.68-2.41)	2.29 (1.88-2.78)	1.77 (1.59-1.97)	1.51 (1.35-1.70)
	**AKI vs. no AKI**		**2.01 (1.68-2.41)**	**2.29 (1.88-2.78)**	**1.77 (1.59-1.97)**	**1.51 (1.35-1.70)**

eGFR 45–59	No AKI	2925	1.14 (1.00-1.30)	1.22 (1.07-1.40)	0.98 (0.91-1.05)	0.97 (0.90-1.04)
	AKI	444	1.51 (1.16-1.97)	1.84 (1.38-2.45)	1.52 (1.33-1.73)	1.21 (1.05-1.39)
	**AKI vs. no AKI**		**1.32 (1.01-1.73)**	**1.50 (1.13-2.00)**	**1.56 (1.37-1.77)**	**1.25 (1.09-1.44)**

eGFR 30–44	No AKI	1359	1.63 (1.38-1.93)	1.71 (1.44-2.02)	1.29 (1.18-1.40)	1.18 (1.08-1.29)
	AKI	374	1.38 (1.01-1.87)	1.61 (1.16-2.24)	1.64 (1.43-1.87)	1.24 (1.07-1.44)
	**AKI vs. no AKI**		**0.84 (0.61-1.16)**	**0.94 (0.68-1.32)**	**1.27 (1.11-1.46)**	**1.05 (0.91-1.22)**

eGFR < 30	No AKI	352	3.78 (2.98-4.80)	3.81 (2.97-4.88)	1.87 (1.62-2.15)	1.65 (1.42-1.90)
	AKI	193	3.36 (2.40-4.69)	3.63 (2.52-5.22)	2.20 (1.85-2.63)	1.57 (1.29-1.90)
	**AKI vs. no AKI**		**0.89 (0.69-1.30)**	**0.95 (0.64-1.41)**	**1.18 (0.96-1.45)**	**0.95 (0.77-1.18)**

Multivariable cause-specific Cox regression with interaction terms between AKI and baseline eGFR. Adjusted estimates are reported with reference to no AKI and eGFR > 60 (plain type), and for AKI versus no AKI within each eGFR group calculated using the interaction terms (bold type). The “fully adjusted” model included adjustment for social, demographic, admission circumstances, each separate nonrenal Charlson comorbidity, and renal measurements as described in the Covariates section.

AKI, acute kidney injury; CI, confidence interval; eGFR, estimated glomerular filtration rate (ml/min per 1.73 m^2^); HR, hazard ratio.

**Table 5:** Relative risk of *de novo* CKD stage 4 after acute kidney injury

Post-hospital episode eGFR	AKI or no AKI	N	Cause-specific new CKD 4; age-sex adjusted (HR, 95% CI)	Cause-specific new CKD 4; fully adjusted (HR, 95% CI)	Competing event of death without renal decline; age-sex adjusted (HR, 95% CI)	Competing event of death without renal decline; fully adjusted (HR, 95% CI)
eGFR ≥ 60	No AKI (reference)	8049	1.00 (reference)	1.00 (reference)	1.00 (reference)	1.00 (reference)
	AKI	955	2.36 (1.31-4.24)	2.55 (1.41-4.64)	1.70 (1.54-1.88)	1.47 (1.32-1.63)
	**AKI vs. no AKI**		**2.36 (1.31-4.24)**	**2.55 (1.41-4.64)**	**1.70 (1.54-1.88)**	**1.47 (1.32-1.63)**

eGFR 45–59	No AKI	2925	7.09 (5.07-9.90)	7.18 (5.14-10.02)	0.93 (0.87-1.00)	0.94 (0.87-1.00)
	AKI	444	10.96 (6.82-17.62)	12.60 (7.63-20.81)	1.46 (1.29-1.65)	1.17 (1.02-1.34)
	**AKI vs. no AKI**		**1.55 (1.02-2.34)**	**1.75 (1.13-2.71)**	**1.56 (1.38-1.77)**	**1.25 (1.09-1.43)**

eGFR 30–44	No AKI	1359	48.54 (35.22-66.91)	50.21 (36.31-69.43)	1.18 (1.08-1.29)	1.10 (1.00-1.20)
	AKI	374	52.40 (36.36-75.52)	61.17 (40.73-91.87)	1.47 (1.28-1.69)	1.12 (0.95-1.31)
	**AKI vs. no AKI**		**1.08 (0.85-1.38)**	**1.22 (0.92-1.61)**	**1.25 (1.08-1.45)**	**1.02 (0.87-1.19)**

Multivariable cause-specific Cox regression with interaction terms between AKI and baseline eGFR. Adjusted estimates are reported with reference to no AKI and eGFR > 60 (plain type) and for AKI versus no AKI within each eGFR group calculated using the interaction terms (bold type). The “fully adjusted” model included adjustment for social, demographic, admission circumstances, each separate nonrenal Charlson comorbidity, and renal measurements as described in the Covariates section.

AKI, acute kidney injury; CI, confidence interval; eGFR, estimated glomerular filtration rate (ml/min per 1.73 m^2^); HR, hazard ratio.

### Sensitivity and subgroup analyses

Supplementary Table 2 shows the interactions tested in further analyses for the renal decline endpoint. The role of AKI (vs. no AKI) was modified by age (interaction *P* value = 0.01) with greater relative risk in the young than in the elderly, but significant in both groups. It was not modified by gender (*P* value = 0.86), diabetes (*P* value = 0.90), cancer (*P* value = 0.78), or cardiac failure (*P* value = 0.23). A statistically significant time interaction (i.e., nonproportionality) was also present (*P* value = 0.04), and therefore in a sensitivity analysis we split follow-up at 5 years after discharge. As reported in Supplementary Table S2, the HR for AKI up to 5 years among those alive at 1 year (1.69, 1.40–2.03) was greater than the HR for AKI up to 10 years for those alive and at risk 5 years after discharge (1.45, 1.12–1.88). As reported in Supplementary Table S3, we also repeated the analysis for AKI versus no AKI excluding those with post-episode proteinuria, with similar pattern results. We also repeated the analysis excluding those who only had a discharge eGFR available for post-episode eGFR, and the findings were unchanged. Further sensitivity analyses included additional adjustment for acute hospital diagnoses (Supplementary Table S4) and reanalysis with the use of a Fine and Gray competing risks model (Supplementary Table S5). Both showed similar results.

## Discussion

This large analysis of hospital survivors after AKI isolates the risk of long-term *subsequent* progression of kidney disease from progression that has already arisen because of an *initial* step drop in kidney function (incomplete recovery). When this novel approach was used, AKI during a hospital admission was associated with increased *subsequent* renal progression irrespective of how progression was defined, irrespective of proteinuria or AKI severity, and even if post-episode kidney function was normal. In one of the longest observation periods of any renal progression study, the excess risk after AKI diminished over time but persisted throughout the 10 years of the study.

Previous studies have also shown an association between AKI and long-term CKD,^1^, ^2^, ^3^, ^4^ but our analysis extends the current understanding by providing greater detail and precision. First, to the best of our knowledge, no previous studies of AKI prognosis have presented renal progression endpoints both defined in terms of an intermediate outcome (30% renal decline) and a hard outcome (*de novo* CKD stage 4 or long-term RRT). This analysis shows that no matter how renal progression is defined, AKI is associated with poorer long-term outcomes, although we recognize that *de novo* CKD stage 4 was most common among those with AKI who already had a low eGFR. Second, previous analyses have used an all-or-nothing “renal recovery” dichotomy as a risk factor for prognostic study in AKI.^8^, ^9^ However, grouping patients in this way does not separate the initial renal decline (i.e., nonrecovery to baseline that is already observable after the episode) from *subsequent* renal decline (the uncertainty of what happens next), nor does it account for intra-episode changes in eGFR that can occur irrespective of AKI.^2^ Our analysis provides the following important detail: whereas 25.7% of people with AKI experienced a 30% decline between their pre-episode and post-discharge eGFR values from the post-discharge eGFR value, 14.8% of people with AKI experienced subsequent renal decline. This represented a relative risk of up to 2.5-fold from AKI (vs. no AKI), which varied depending on the level of post-episode eGFR.

The interaction between AKI and post-episode eGFR is a unique finding of this analysis. The increased relative risk from AKI (vs. no AKI) was greatest (more than 2-fold) among those who experienced recovery to normal levels (eGFR ≥ 60), even when those with post-episode proteinuria were excluded. This finding is in contrast to the Kidney Disease: Improving Global Outcomes AKI guidelines, which recommend prioritization of those with *de novo* CKD at a 90- day clinical reassessment.^7^ However, regarding the lack of elevated relative risk from AKI among those with a low post-episode eGFR, we note for the reader that *subsequent* decline represents only one mode of progression. Indeed, a step 30% eGFR drop during admission was particularly common among those with AKI and low post-episode eGFR, indicating that nonrecovery with low subsequent progression was common. The interaction between AKI and eGFR on progression also complements previous studies, which have demonstrated a similar interaction between AKI and eGFR on mortality.^11^, ^12^ Finally, a complementary explanation for the poorer outcomes among those with a normal post-episode eGFR could be that AKI indicates a “failed stress test” unmasking subclinical renal disease. This is biologically plausible because some people with ostensibly “normal” kidney function nevertheless lack functional glomerular filtration reserve.^13^ The important clinical implication is that such patients will be more vulnerable to future decline, even though currently available metrics of renal function remain “normal.” In this situation a recent AKI episode yields important prognostic information that may otherwise be overlooked if not clearly communicated at any transitions in care.

A strength of this analysis is the use of routinely collected data within a large regional population with long follow-up. This “real-life” situation maximizes the generalizability of our findings. Another strength is the distinction of 2 perspectives on progression by using both intermediate (30% renal decline) and hard (CKD stage 4) endpoints. Moreover, by defining the study entry eGFR at a post-discharge time point, our analysis provides a precise separation of incomplete initial recovery and *subsequent* renal decline that has not previously been studied. A limitation is that data collection was not protocolized. This means there may be ascertainment biases in our determination of renal progression, but these will have been partially offset by our requirement for the outcomes to be sustained for at least 90 days. We also conducted a sensitivity analysis excluding patients with only a discharge creatinine value available in the first post-episode year, with unchanged results. Similarly, quantified proteinuria was frequently not tested in the cohort. In most cases this will have been because of a low level of suspicion for proteinuria or a negative urinalysis, but some cases of proteinuria may have been missed. We also recognize that post-AKI proteinuria will frequently reflect underlying renal disease rather than a new derangement consequent to AKI. Finally, as with all observational studies, there will be residual confounding, which means that the long-term role of AKI may have been overestimated. Our analyses included adjustment for confounders including social, demographic, and renal measurements; comorbid factors; and acute diagnoses in a sensitivity analysis. However, we recognize that hospital episode International Classification of Diseases, 10th Revision (ICD-10) codes for comorbidities may lack granularity. We note that prospective recruitment and protocolized follow-up could overcome some of these issues but would be at the expense of “real-life” generalizability.

Overall, this study shows that no matter how severe an AKI episode is, irrespective of proteinuria and even if post-episode function is apparently preserved, an episode of AKI is associated with increased subsequent renal decline that persists for up to 10 years. Recommendations for follow-up should therefore be formulated carefully to avoid false reassurance when eGFR after AKI appears to have returned to the normal range.

## Methods

### Population

The Grampian Laboratory Outcomes Morbidity and Mortality Study^12^, ^14^, ^15^ is a population cohort achieved by linking national and regional data sources for a single United Kingdom health authority (Grampian resident adult population 438,332).^16^ Nonresidents have been excluded. Because data linkage avoids the need for active recruitment, this virtual cohort is not affected by the selection biases inherent in patient enrollment. The region includes a large tertiary center (∼1000 beds) and 2 outlying hospitals (combined ∼500 beds). All biochemistry testing was provided by a single biochemistry service (1999–2013), regardless of clinical location (inpatient, outpatient, community). This minimizes any loss of baseline and follow-up data, which are vital in renal clinical research.^17^ Information on mortality, hospital admission episodes, morbidity events, and long-term RRT are available by linkage to hospital episode data, the local renal information management system, and the Scottish Renal Registry.

### Study entry

This study includes patients from the Grampian Laboratory Outcomes Morbidity and Mortality Study who survived to the completion of a hospital admission episode in 2003.^12^ We chose 1 year after discharge as the time point when the index episode was considered complete and subjects “entered” the study. The most recent eGFR available at study entry (1 year after an episode) was taken as the reference value for determining all subsequent renal outcomes. For the rest of this article, we refer to this as the “post-episode eGFR.” Those who were dead or receiving long-term RRT at study entry were excluded.

We selected “1 year after episode” to optimize opportunity for renal recovery and a completeness of post-episode testing. This testing was performed a median 264 days after discharge. As shown in Supplementary Table S1, had study entry been earlier, post-episode tests would have been unavailable in 39% of patients with AKI and 55% of patients who did not have AKI. For the minority of patients who still did not have a test at 1 year, the eGFR at discharge was used as the reference. These patients were also removed in a sensitivity analysis to ensure this did not affect the results.

### Exposure

AKI during the index hospital admission was identified and staged from 1 to 3 by using AKI criteria based on the Kidney Disease: Improving Global Outcomes (KDIGO) Acute Kidney Injury Work Group guidelines.^7^ Baseline creatinine values for identifying AKI were determined by a modified “e-alert” algorithm with a hierarchy of criteria for creatinine changes from the previous 48 hours and 7, 90, and 365 days as stated elsewhere.^12^ Briefly, this definition for AKI involves 1 of 3 criteria: serum creatinine level ≥ 1.5 times higher than the median of all creatinine values 8 to 90 days earlier, or 91 to 365 days earlier if no tests were done between 8 and 90 days; serum creatinine level ≥ 1.5 times higher than the lowest creatinine value within 7 days; or serum creatinine level > 26 μmol/l higher than the lowest creatinine value within 48 hours. We have previously described this definition in more detail for studying the prognosis of AKI in hospitals.^12^

### Outcomes

Two subsequent renal progression outcomes are defined for this analysis: sustained 30% eGFR decline (in all 14,651 patients) and *de novo* CKD stage 4 (in 14,106 patients with post-episode eGFR ≥ 30 ml/min per 1.73 m^2^). For either outcome, progression was sustained if it lasted at least 90 days or if the patient started long-term RRT. Notably, as illustrated in Supplementary Table S1, if a pre-episode eGFR (instead of the post-episode eGFR) had been used as reference value for subsequent renal outcomes, 25.7% of patients with AKI would already have had a 30% eGFR decline on the first day of the study (i.e., nonrecovery from AKI misclassified as post-episode renal decline).

### Follow-up

Follow-up began at study entry, 1 year after discharge from the index hospital episode. It lasted until the date of renal progression (respectively sustained 30% renal decline or new CKD stage 4 for each progression subanalysis), death, or the end of study follow-up in July 2013.

### Covariates

We adjusted for all other observable renal measurements at study entry to isolate the post-episode role of an AKI episode. These included the most recent post-episode eGFR available at study entry, the “intra-episode background change in eGFR,” the presence of any other prior AKI episodes in the previous 3 years, and quantified proteinuria during the 1-year post-episode period.

We defined “intra-episode background change in eGFR” as the difference between the pre-episode baseline eGFR and the post-episode eGFR (Figure 1). The reason for adjusting for this change in addition to all other renal measurements is that background rise or fall in eGFR can occur during any admission episode, irrespective of AKI (e.g., because of loss of muscle mass or slow background renal progression).

All eGFR measures were reported using the Chronic Kidney Disease Epidemiology Collaboration creatinine equation.^18^ We grouped post-episode eGFR in 4 categories: ≥60, 45–59, 30–44, and <30 ml/min per 1.73 m^2^. To allow for nonlinearity, we also grouped intra-episode eGFR change in 5 categories: >30% rise, 10%–30% rise, no change, 10%–30% decline, and >30% decline.^19^ Proteinuria measures recorded as “abnormal” were albumin creatinine ratio ≥ 3 mg/mmol or protein creatinine ratio ≥ 15 mg/mmol.^20^

Nonrenal comorbidities were determined using ICD-10 codes for Charlson comorbidities from the 5 years before admission as previously described and validated.^21^ Social and demographic measures included age, sex, whether the patient was in residential care, and home address–based measures of deprivation (most deprived quintile versus the other 4 quintiles of the Scottish Index of Multiple Deprivation), and rural location (settlement of less than 3000 people).^22^ Metrics of admission circumstances were whether the index hospital admission was an emergency or elective and whether the index admission included a stay in a medical (vs. surgical) ward or a critical care or intensive care unit.

### Statistical analyses

For both renal progression outcomes (first for 30% eGFR decline, then for *de novo* CKD stage 4), we reported patient characteristics grouped as follows: those who were alive without renal progression at the end of follow-up, those who experienced renal progression, and those who died without experiencing renal progression (a competing risk).

We plotted crude outcomes both for 30% renal decline and de novo CKD stage 4 during follow-up by AKI and eGFR category. We also estimated and plotted the cumulative incidence of renal progression, accounting for the competing risk of death using the Stata command “stcompet” with Stata SE 13.0 software (StataCorp LLC, College Station, TX) as described elsewhere.^23^

We estimated the independent association of AKI (vs. no AKI) with long-term renal progression in multivariable analysis using both cause-specific Cox models for progression and death without progression. The fully adjusted model included adjustment for social, demographic, admission circumstances, each separate nonrenal Charlson comorbidity, and renal measurements as described previously in the Covariates section. Because an interaction exists between eGFR (in categories ≥60, 45–59, 30–45, <30) and AKI (vs. no AKI) on mortality,^12^, ^24^ we included an eGFR AKI interaction term for renal progression in this analysis. All analyses were conducted in Stata SE 13.0 software (StataCorp LLC).

### Subgroup and sensitivity analyses

We tested for interactions of AKI and progression with old age (≥70 years), sex, cancer, and diabetes. We also tested an interaction with follow-up time (per year of follow-up completed) to assess the proportionality assumption for AKI. In the main analysis, we presented cause-specific HRs for individuals alive and at risk, which is the preferred approach for estimating the effect of covariates on outcomes as HRs.^25^ However, in sensitivity analyses, we also estimated “subdistribution HRs” according to the Fine and Gray model (in which those who died without progression remain in the “risk set”).^25^, ^26^ Because it is not possible to distinguish whether acute diagnoses recorded during the index admission were a cause or consequence of AKI, we did not adjust for acute diagnoses in the primary multivariable analysis, but in a sensitivity analysis, we compared the findings after acute hospital diagnoses (extracted from ICD-10 codes) were added to the models.^12^, ^27^ Because the most recent available post-episode eGFR was at discharge for 20% of the patients, we also repeated the analysis excluding these patients. Finally, because it is possible for patients to have a normal eGFR (≥ 60 ml/min per 1.73 m^2^) but still have proteinuric evidence of nonrecovery,^20^ we repeated the analysis excluding those with abnormal proteinuria measurements.

## Disclosure

All the authors declared no competing interests.

## Supplementary Material

Figure S1 Crude long-term renal outcomes up to 5 years after a hospital admission episode with or without acute kidney injury (AKI).

Table S1 Post-episode estimated glomerular filtration rate in the first year after discharge from the index admission episode.

Table S2 Analyses of interactions.

Table S3 Relative risk of renal progression after excluding those with post-episode proteinuria.

Table S4 Relative risk of subsequent sustained 30% renal decline and new chronic kidney disease stage 4 after acute kidney injury with additional adjustment for acute hospital diagnoses.

Table S5 Relative risk of subsequent sustained 30% renal decline and new chronic kidney disease stage 4 after acute kidney injury using Fine and Gray model.
