# Supplementary material for: Post-discharge kidney function is associated with subsequent ten-year renal progression risk among survivors of acute kidney injury
Source: Kidney Int. 2017 Aug;92(2):440–52. doi: 10.1016/j.kint.2017.02.019 (PMC5524434; doi:10.1016/j.kint.2017.02.019)
Supplement: Figure S1 — Crude long-term renal outcomes up to 5 years after a hospital admission episode with or without acute kidney injury (AKI). [file mmc1.ppt]

## Slide 1
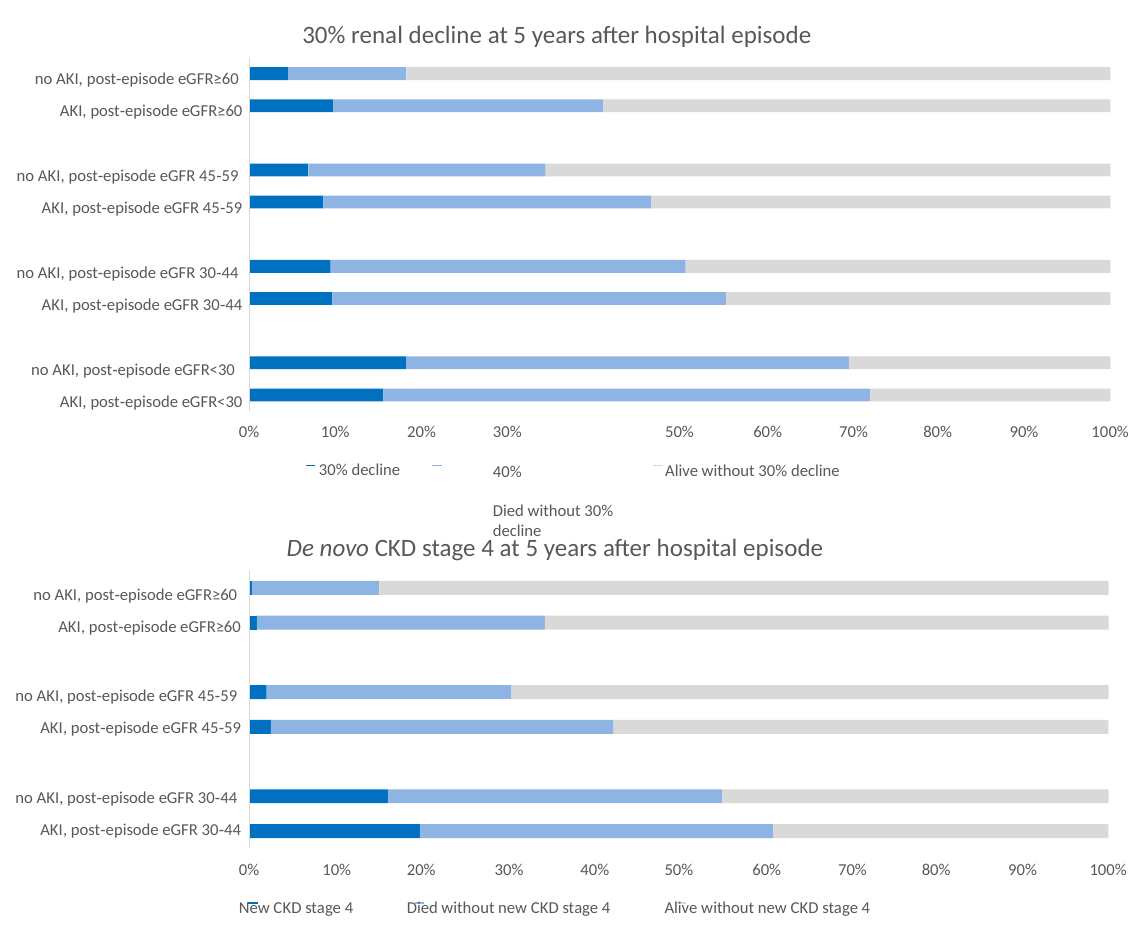

30% renal decline at 5 years after hospital episode
no AKI, post‐episode eGFR≥60 AKI, post‐episode eGFR≥60
no AKI, post‐episode eGFR 45‐59 AKI, post‐episode eGFR 45‐59
no AKI, post‐episode eGFR 30‐44 AKI, post‐episode eGFR 30‐44
no AKI, post‐episode eGFR<30
AKI, post‐episode eGFR<30
0%
10%
20%
30%	40%
Died without 30% decline
50%	60%	70%
Alive without 30% decline
80%
90%
100%
30% decline
De novo CKD stage 4 at 5 years after hospital episode
no AKI, post‐episode eGFR≥60 AKI, post‐episode eGFR≥60
no AKI, post‐episode eGFR 45‐59 AKI, post‐episode eGFR 45‐59
no AKI, post‐episode eGFR 30‐44 AKI, post‐episode eGFR 30‐44
0%	10%
New CKD stage 4
20%	30%	40%
Died without new CKD stage 4
50%	60%	70%
Alive without new CKD stage 4
80%
90%
100%
