# Supplementary material for: Post-discharge kidney function is associated with subsequent ten-year renal progression risk among survivors of acute kidney injury
Source: Kidney Int. 2017 Aug;92(2):440–52. doi: 10.1016/j.kint.2017.02.019 (PMC5524434; doi:10.1016/j.kint.2017.02.019)
Supplement: Table S1 — Post-episode estimated glomerular filtration rate in the first year after discharge from the index admission episode. [file mmc2.docx]

Supplementary table 1 – Post-episode eGFR in the first year after discharge from the index admission episode

|  | AKI patients | | | | | | | | | |  | Non-AKI patients | | | | | | | | | | |  |
| --- | --- | --- | --- | --- | --- | --- | --- | --- | --- | --- | --- | --- | --- | --- | --- | --- | --- | --- | --- | --- | --- | --- | --- |
|  | **Discharge  (0 days)** | | **1-90 days** | | **91-180 days** | | **181-270 days** | | **271-365 days** | |  | **Discharge  (0 days)** | | **1-90 days** | | **91-180 days** | | **181-270 days** | | **271-365 days** | |  |  |
| **Change in eGFR compared to  pre-episode baseline eGFR  (in categories)** |  |  |  |  |  |  |  |  |  |  |  |  |  |  |  |  |  |  |  |  |  |  |  |
| >30% rise | 81 | (4.12) | 87 | (4.43) | 77 | (3.92) | 71 | (3.61) | 67 | (3.41) |  | 877 | (6.91) | 1013 | (7.99) | 1070 | (8.44) | 1086 | (8.56) | 1068 | (8.42) |  |  |
| 10-30% rise | 157 | (7.99) | 144 | (7.32) | 125 | (6.36) | 128 | (6.51) | 120 | (6.10) |  | 1321 | (10.41) | 1709 | (13.47) | 1887 | (14.88) | 1899 | (14.97) | 1949 | (15.36) |  |  |
| no change | 492 | (25.03) | 555 | (28.23) | 548 | (27.87) | 550 | (27.98) | 517 | (26.30) |  | 9775 | (77.06) | 8566 | (67.53) | 7886 | (62.17) | 7500 | (59.12) | 7137 | (56.26) |  |  |
| 10-30% decline | 688 | (34.99) | 733 | (37.28) | 748 | (38.05) | 725 | (36.88) | 757 | (38.50) |  | 676 | (5.33) | 1302 | (10.26) | 1675 | (13.20) | 1967 | (15.51) | 2233 | (17.60) |  |  |
| >30% decline | 548 | (27.87) | 447 | (22.74) | 468 | (23.80) | 492 | (25.03) | 505 | (25.69) |  | 36 | (0.28) | 95 | (0.75) | 167 | (1.32) | 233 | (1.84) | 298 | (2.35) |  |  |
|  |  |  |  |  |  |  |  |  |  |  |  |  |  |  |  |  |  |  |  |  |  |  |  |
| **Post-episode monitoring** |  |  |  |  |  |  |  |  |  |  |  |  |  |  |  |  |  |  |  |  |  |  |  |
| post-episode test unavailable | na | (-) | 763 | (38.56) | 500 | (25.43) | 384 | (19.53) | 289 | (14.70) |  | na | (-) | 7026 | (55.39) | 4892 | (38.57) | 3749 | (29.55) | 3011 | (23.74) |  |  |
| repeat test taken during period | na | (-) | 1203 | (61.19) | 1011 | (51.42) | 903 | (45.93) | 904 | (45.98) |  | na | (-) | 5659 | (44.61) | 4890 | (38.55) | 4525 | (35.67) | 4581 | (36.11) |  |  |
|  |  |  |  |  |  |  |  |  |  |  |  |  |  |  |  |  |  |  |  |  |  |  |  |
| **Re-categorisation compared to  previous period** |  |  |  |  |  |  |  |  |  |  |  |  |  |  |  |  |  |  |  |  |  |  |  |
| improvement | na | (-) | 376 | (19.13) | 213 | (10.83) | 177 | (9.00) | 165 | (8.39) |  | na | (-) | 1174 | (9.26) | 1006 | (7.93) | 923 | (7.28) | 958 | (7.55) |  |  |
| deterioration | na | (-) | 282 | (14.34) | 308 | (15.67) | 227 | (11.55) | 239 | (12.16) |  | na | (-) | 1312 | (10.34) | 1218 | (9.60) | 1237 | (9.75) | 1277 | (10.07) |  |  |
| no change in category | na | (-) | 1308 | (66.53) | 1445 | (73.50) | 1562 | (79.45) | 1562 | (79.45) |  | na | (-) | 10199 | (80.40) | 10461 | (82.47) | 10525 | (82.97) | 10450 | (82.38) |  |  |
| Abbreviations: AKI, acute kidney injury; eGFR, estimated glomerular filtration rate; na, not applicable. | | | | | | | | | | | | | | | | | | | | | | | |
