# Supplementary material for: Post-discharge kidney function is associated with subsequent ten-year renal progression risk among survivors of acute kidney injury
Source: Kidney Int. 2017 Aug;92(2):440–52. doi: 10.1016/j.kint.2017.02.019 (PMC5524434; doi:10.1016/j.kint.2017.02.019)
Supplement: Table S2 — Analyses of interactions. [file mmc3.docx]

Supplementary table 2 – Analyses of interactions

| **30% renal decline outcome  (sensitivity analyses of interactions)** | **N** | **Cause specific renal decline;  fully adjusted (HR, 95% CI)** | |
| --- | --- | --- | --- |
| AKI vs no AKI; among age<70 | 7622 | 2.29 | (1.83-2.87) |
| AKI vs no AKI; among age≥70 | 7029 | 1.45 | (1.21-1.73) |
| **interaction p value** |  | **0.01** |  |
| AKI vs no AKI; among female | 8317 | 1.66 | (1.37-2.01) |
| AKI vs no AKI; among male | 6334 | 1.70 | (1.39-2.09) |
| **interaction p value** |  | **0.86** |  |
| AKI vs no AKI; among no diabetes | 13734 | 1.68 | (1.42-1.97) |
| AKI vs no AKI; among diabetes | 917 | 1.71 | (1.24-2.36) |
| **interaction p value** |  | **0.90** |  |
| AKI vs no AKI; among no cancer | 13640 | 1.69 | (1.45-1.97) |
| AKI vs no AKI; among cancer | 1011 | 1.58 | (1.02-2.47) |
| **interaction p value** |  | **0.78** |  |
| AKI vs no AKI; among no cardiac failure | 13983 | 1.73 | (1.48-2.02 |
| AKI vs no AKI; among cardiac failure | 668 | 1.29 | (0.82-2.03) |
| **interaction p value** |  | **0.23** |  |
| AKI vs no AKI for those at risk between 1 and 5 years | 14651 | 1.69 | (1.40-2.03) |
| AKI vs no AKI for those at risk between 5 and 10 years | 10302 | 1.45 | (1.12-1.88) |
| **time interaction p value** |  | **0.04** |  |
| Note: The “fully-adjusted” model included adjustment for social, demographic, admission circumstances, each separate non-renal Charlson comorbidity and renal measurements as described in the “covariates” section.  Abbreviations: AKI, acute kidney injury; CI, confidence interval; eGFR, estimated glomerular filtration rate (ml/min/1.73m^2^); HR, hazard ratio. | | | |
