# Supplementary material for: Post-discharge kidney function is associated with subsequent ten-year renal progression risk among survivors of acute kidney injury
Source: Kidney Int. 2017 Aug;92(2):440–52. doi: 10.1016/j.kint.2017.02.019 (PMC5524434; doi:10.1016/j.kint.2017.02.019)
Supplement: Table S3 — Relative risk of renal progression after excluding those with post-episode proteinuria. [file mmc4.docx]

Supplementary table 3 – Relative risk of renal progression after excluding those with post-episode proteinuria

|  | **Cause specific renal decline;  including all at risk (HR, 95% CI)** | | **Cause specific renal decline;  excluding those with proteinuria (HR, 95% CI)** | | **Cause specific *de novo* CKD stage 4;  including all at risk (HR, 95% CI)** | | **Cause specific *de novo* CKD stage 4;  excluding those with proteinuria (HR, 95% CI)** | |
| --- | --- | --- | --- | --- | --- | --- | --- | --- |
| **AKI vs no AKI, eGFR≥60** | **2.29** | **(1.88-2.78)** | **2.33** | **(1.89-2.87)** | **2.55** | **(1.41-4.64)** | **2.15** | **(1.08-4.28)** |
| **AKI vs no AKI, eGFR 45-59** | **1.50** | **(1.13-2.00)** | **1.53** | **(1.12-2.11)** | **1.75** | **(1.13-2.71)** | **1.75** | **(1.07-2.85)** |
| **AKI vs no AKI, eGFR 30-44** | **0.94** | **(0.68-1.32)** | **1.01** | **(0.68-1.50)** | **1.22** | **(0.92-1.61)** | **1.35** | **(0.99-1.85)** |
| **AKI vs no AKI, eGFR<30** | **0.95** | **(0.64-1.41)** | **0.60** | **(0.32-1.13)** | **n/a** |  | **n/a** |  |
| Note: The “fully-adjusted” model included adjustment for social, demographic, admission circumstances, each separate non-renal Charlson comorbidity and renal measurements as described in the “covariates” section.  Abbreviations: AKI, acute kidney injury; CI, confidence interval; eGFR, estimated glomerular filtration rate (ml/min/1.73m^2^); HR, hazard ratio. | | | | | | | | |
