# Supplementary material for: Post-discharge kidney function is associated with subsequent ten-year renal progression risk among survivors of acute kidney injury
Source: Kidney Int. 2017 Aug;92(2):440–52. doi: 10.1016/j.kint.2017.02.019 (PMC5524434; doi:10.1016/j.kint.2017.02.019)
Supplement: Table S4 — Relative risk of subsequent sustained 30% renal decline and new chronic kidney disease stage 4 after acute kidney injury with additional adjustment for acute hospital diagnoses. [file mmc5.docx]

Supplementary table 4 – Relative risk of subsequent sustained 30% renal decline and new CKD stage 4 after AKI with additional adjustment for acute hospital diagnoses

| **Post hospital episode eGFR** | **AKI or no AKI** | **N** | **Cause specific renal decline;  fully adjusted (HR, 95% CI)** | | **Cause specific new CKD stage 4;  fully adjusted (HR, 95% CI)** | |
| --- | --- | --- | --- | --- | --- | --- |
|  |  |  |  |  |  |  |
| eGFR≥60 | No AKI (reference) | 8049 | 1.00 | (reference) | 1.00 | (reference) |
|  | AKI | 955 | 1.97 | (1.62-2.40) | 2.26 | (1.25-4.11) |
|  | **AKI vs no AKI** |  | **1.97** | **(1.62-2.40)** | **2.26** | **(1.25-4.11)** |
|  |  |  |  |  |  |  |
| eGFR 45-59 | No AKI | 2925 | 1.31 | (1.13-1.51) | 7.47 | (5.18-10.78) |
|  | AKI | 444 | 1.76 | (1.31-2.37) | 11.88 | (7.04-20.03) |
|  | **AKI vs no AKI** |  | **1.35** | **(1.01-1.80)** | **1.59** | **(1.02-2.47)** |
|  |  |  |  |  |  |  |
| eGFR 30-44 | No AKI | 1359 | 1.67 | (1.39-2.01) | 46.69 | (32.46-67.14) |
|  | AKI | 374 | 1.47 | (1.05-2.05) | 52.85 | (34.16-81.79) |
|  | **AKI vs no AKI** |  | **0.88** | **(0.63-1.24)** | **1.13** | **(0.85-1.52)** |
|  |  |  |  |  |  |  |
| eGFR<30 | No AKI | 352 | 2.87 | (2.19-3.76) | - | Not applicable |
|  | AKI | 193 | 2.81 | (1.91-4.12) | - | Not applicable |
|  | **AKI vs no AKI** |  | **0.98** | **(0.65-1.47)** | **-** | **Not applicable** |
| Note: Multivariable cause specific Cox regression with interaction terms between AKI and baseline eGFR. Adjusted estimates are reported with reference to no AKI and eGFR>60 (plain type), and for AKI vs no AKI within each eGFR group calculated using the interaction terms (bold type). The “fully-adjusted” model included adjustment for social, demographic, admission circumstances, each separate non-renal Charlson comorbidity and renal measurements as described in the “covariates” section and acute hospital diagnoses.  Abbreviations: AKI, acute kidney injury; CI, confidence interval; eGFR, estimated glomerular filtration rate (ml/min/1.73m^2^); HR, hazard ratio; SHR. | | | | | | |
