# Supplementary material for: Post-discharge kidney function is associated with subsequent ten-year renal progression risk among survivors of acute kidney injury
Source: Kidney Int. 2017 Aug;92(2):440–52. doi: 10.1016/j.kint.2017.02.019 (PMC5524434; doi:10.1016/j.kint.2017.02.019)
Supplement: Table S5 — Relative risk of subsequent sustained 30% renal decline and new chronic kidney disease stage 4 after acute kidney injury using Fine and Gray model. [file mmc6.docx]

Supplementary table 5 – Relative risk of subsequent sustained 30% renal decline and new CKD stage 4 after AKI using Fine and Gray model

| **Post hospital episode eGFR** | **AKI or no AKI** | **N** | **Fine and Gray model for renal decline;  fully adjusted (SHR, 95% CI)** | | **Fine and Gray model for new CKD stage 4;  fully adjusted (SHR, 95% CI)** | |
| --- | --- | --- | --- | --- | --- | --- |
|  |  |  |  |  |  |  |
| eGFR≥60 | No AKI (reference) | 8049 | 1.00 | (reference) | 1.00 | (reference) |
|  | AKI | 955 | 2.21 | (1.81-2.69) | 2.52 | (1.39-4.58) |
|  | **AKI vs no AKI** |  | **2.21** | **(1.81-2.69)** | **2.52** | **(1.39-4.58)** |
|  |  |  |  |  |  |  |
| eGFR 45-59 | No AKI | 2925 | 1.21 | (1.05-1.38) | 7.14 | (5.11-9.97) |
|  | AKI | 444 | 1.71 | (1.28-2.28) | 12.36 | (7.45-20.51) |
|  | **AKI vs no AKI** |  | **1.41** | **(1.06-1.88)** | **1.73** | **(1.12-2.69)** |
|  |  |  |  |  |  |  |
| eGFR 30-44 | No AKI | 1359 | 1.65 | (1.39-1.96) | 49.64 | (35.85-68.75) |
|  | AKI | 374 | 1.45 | (1.04-2.02) | 53.58 | (38.69-88.68) |
|  | **AKI vs no AKI** |  | **0.88** | **(0.63-1.23)** | **1.18** | **(0.89-1.57)** |
|  |  |  |  |  |  |  |
| eGFR<30 | No AKI | 352 | 3.72 | (2.90-4.79) | - | Not applicable |
|  | AKI | 193 | 3.27 | (2.26-4.74) | - | Not applicable |
|  | **AKI vs no AKI** |  | **0.88** | **(0.60-1.31)** | **-** | **Not applicable** |
| Note: Fine and Gray model with interaction terms between AKI and baseline eGFR. Adjusted estimates are reported with reference to no AKI and eGFR>60 (plain type), and for AKI vs no AKI within each eGFR group calculated using the interaction terms (bold type). The “fully-adjusted” model included adjustment for social, demographic, admission circumstances, each separate non-renal Charlson comorbidity and renal measurements as described in the “covariates” section.  Abbreviations: AKI, acute kidney injury; CI, confidence interval; eGFR, estimated glomerular filtration rate (ml/min/1.73m^2^); SHR, subdistribution hazard ratio; SHR. | | | | | | |
